# Author's reply

**DOI:** 10.1192/bjb.2018.34

**Published:** 2018-06

**Authors:** Joanna Moncrieff

**Affiliations:** University College London; email: j.moncrieff@ucl.ac.uk

In response to Dr Jauhar and Professor Young, I am used to being accused of using ideology, of being selective, of not being balanced or of being polemical. I take no personal offence, but it is important to point out that this is a useful tactic if you want to shut down debate. It harnesses the authority of science to present one view as neutral, objective and credible, and the other as self-interested and unreliable. In truth, we all bring assumptions and biases to our work. I am obviously unable to describe every study ever done on antidepressants in a short article, but I have written books and papers that address all the evidence I could find that supports the disease-centred model of drug action in relation to antidepressants and other psychiatric drugs.^1^

Indeed, one of the most important points I am making in relation to drug action is that existing psychopharmacological research is based on unexamined assumptions about how drugs work. These consist of the idea that drugs target the neurological mechanisms underlying symptoms, whether the latest theory about mechanisms concerns abnormalities of neurotransmitters, neural networks or neuro-plasticity. This idea has allowed psychopharmacology research to ignore the alterations to normal functioning that psychiatric drugs produce, and that will affect mental states including mental disorders, regardless of the underlying mechanisms.

Jauhar and Young point out that the latest meta-analysis of antidepressant trials finds impressive odds ratios for effects of antidepressants, but it analyses categorical outcomes derived from continuous data, which has been shown to inflate drug–placebo differences.^2^ Network analysis is also likely to exaggerate differences, since the degree of improvement in comparative trials is higher than in placebo-controlled trials.^3^ The continuous data, which showed a standardised mean difference (SMD) of 0.3, is in line with other meta-analyses in showing small and almost certainly clinically insignificant differences between antidepressants and placebo, equivalent to around two points difference on the Hamilton Rating Scale for Depression (HRSD).^4^^,^^5^

Jauhar and Young make a good point about the validity of the HRSD, but it is nevertheless used as the primary outcome of most trials. Analyses of the subjective mood item are thus more likely to be influenced by selective reporting of positive findings. However, they are wrong about the Medical Research Council trial, where the dose of imipramine was 200 mg and that of phenelzine was 60 mg. There is indeed evidence either way on the association between severity and antidepressant response, but even in studies with positive findings, effect sizes in those with severe depression are small and unlikely to be clinically significant, and the association may be accounted for by differing expectations in people with more or less severe symptoms.^6^

I agree with the gist of Dr Dunleavy's response. Recommending antidepressants because they produce emotion-blunting effects, or other useful mental alterations (sedation with tricyclics, for example), is a drug-centred model of prescribing. I don't have a problem with this as long as the patient is properly informed that placebo-controlled trials suggest little if any superiority of antidepressants, that they have full knowledge of all the potential adverse effects, and that they are quite clear that the idea that antidepressants correct an underlying chemical imbalance is not supported by evidence. Then they can make their own informed decision.
